# Zinc Translocation from Coastal Soil to Wheat as Mediated by Zinc Supply Levels and Soil Properties

**DOI:** 10.3390/plants14131971

**Published:** 2025-06-27

**Authors:** Deyong Zhao, Jie Dong, Yan Li

**Affiliations:** 1 College of Biological and Pharmaceutical Engineering, Shandong University of Aeronautics, Binzhou 256603, China; dongjie@stu.sdua.edu.cn (J.D.); 23021201005@sdua.edu.cn (Y.L.); 2 Shandong Key Laboratory of Eco-Environmental Science for Yellow River Delta, Shandong University of Aeronautics, Binzhou 256603, China; 3 Shandong Engineering and Technology Research Center for Fragile Ecological Belt of Yellow River Delta, Binzhou 256603, China

**Keywords:** soil properties, Zn translocation, soil–wheat system, Zn biofortification, coastal saline soil

## Abstract

The association between soil properties and zinc (Zn) availability, as well as how soil properties affect the Zn translocation from coastal soil to wheat grain, was not well understood. A pot study and field trial were conducted to examine the effects of soil properties and Zn application on grain yield and grain Zn concentration (Zn-conc) in wheat grown under coastal soils. Soil DTPA-Zn content positively correlated with concentrations of total Zn, total P, Olsen-P, and ammonia-N in soil. Zn-conc in aboveground plants negatively correlated with soil pH and Olsen-P. Total Zn accumulation (Zn-acc) in aboveground plants varied greatly among different soil treatments. Zn-acc positively correlated with soil DTPA-Zn content, Zn-conc in aboveground plants, aboveground biomass, and root weight. PLS-PM model analysis suggested that soil Zn supply and plant growth had direct effects on Zn utilization in wheat, while soil properties, soil nutrients, and soil available nutrients had indirect effects on Zn utilization in wheat by affecting soil Zn supply and/or plant growth. Grain yield and grain Zn-conc were increased by Zn application under low soil salinity, while Zn application under higher soil salinity did not increase grain Zn-conc. Soil Zn application increased both grain yield and grain Zn-conc of 20 wheat genotypes, while foliar Zn application further increased the average grain Zn-conc without an increase in grain yield. Adjusting the Zn supply tailored to suitable genotypes according to soil properties is promising to reach the Zn biofortification target and a satisfactory wheat grain yield under coastal saline soils.

## 1. Introduction

Soil zinc (Zn) availability affects not only the grain yield but also grain Zn-conc, a nutritional quality index of the crop. About 17% of the global population has an insufficient intake of Zn, an essential micronutrient for maintaining human health [1]. Achieving the Zn biofortification target in wheat is of great significance in reducing Zn deficiency-related diseases, as wheat grain is an important source of Zn nutrition for humans. The Zn-conc in wheat grain under most fields, however, is below the proposed biofortification target of 40 mg kg^−1^ [2,3]. The translocation of Zn from soil to grain is affected by intrinsic genetic factors of crop genotype, soil properties, and agronomic practices. Correspondingly, breeding and agronomic management are two approaches that could offer potential approaches toward increasing the Zn-conc of wheat grain. Genetic variation among genotypes provides opportunities for genetic improvement by traditional breeding and/or the manipulation of genes responsible for Zn transport is promising to increase grain Zn content [4,5,6,7,8]. On the other hand, an agronomic approach such as Zn fertilizer management (foliar and/or soil application) and inoculation of Zn-solubilizing bacteria can have positive effects on improving grain Zn-conc [9,10,11].

The total Zn content in soil varies largely, from 2 to 3548 mg kg^−1^, with a mean of 64 mg kg^−1^ globally [12]. Different forms of Zn are present in soil, i.e., Zn^2+^, ZnOH^+^, and soluble organic Zn, which are assumed to be easily taken up by plants [12]. The Zn fraction extracted by Diethylene Triamine Penta Acetic acid (DTPA) [13] or Mehlich No. 3 [14] is often used to indicate the available Zn in agricultural systems. The Zn availability in soil could be affected by soil properties such as soil pH, organic matter content, and soil texture. Soil pH is a key factor affecting solubility, ionic form, adsorption, and the mobility of plant nutrients, including Zn [15,16]. Zn availability tends to be high in soils with a neutral pH. Low Zn availability can occur in acid soils (pH 5–6.5) and in environments with a higher pH [3,12]. Soil organic matter also affects Zn translocation in the soil–crop system. Increased soil organic matter may lead to the increased adsorption of Zn, hence reducing Zn availability [17]. On the other hand, the Zn availability may be increased with increased soil organic matter content due to the formation of soluble organic Zn complexes or soil organic matter mineralization [18,19]. So, whether the increased soil organic content can result in higher soil Zn availability depends on the interaction between the soil and added soil organic matter. In general, a moderate addition of organic matter into soil can increase the grain Zn content [20], but high content (>3%) of organic matter can also cause Zn deficiency in crops [12]. A recent study showed that Zn availability was mostly driven by soil properties rather than by fertilization [21]. Thus, a balance between organic content with soil Zn status [22], together with considering soil properties, is required for reaching a higher grain yield and/or grain Zn-conc. Soil texture can affect Zn availability by influencing the water-holding capacity and aeration of soil, as well as the adsorption and desorption of Zn. A survey conducted in Europe indicated that clay content explained the most variation in the overall distribution of soil Zn, and coarser soil was associated with lower Zn concentrations [23].

Other ions in soil, such as calcium, iron, and phosphorus, can compete with Zn for uptake by plants, leading to reduced Zn availability. In particular, high levels of phosphorus in soil can inhibit Zn uptake by plants. The possible soil and biological mechanisms for this includes (1) soluble phosphorus fractions in soil reacting with Zn^2+^ to form insoluble Zn_3_(PO_4_)_2_, reducing the availability of both phosphorus and Zn [24]; (2) Zn_3_(PO_4_)_2_ may also be generated in the root system, which limits the transport of zinc from root to shoot and grain; and (3) a higher phosphorus application dose leads to changes in soil pH, arbuscular mycorrhizal colonization, root exudates, and the spatiotemporal expression of Zn transporters, reducing the availability of Zn and phosphorus [25,26,27,28]. The application of nitrogen fertilizer at a proper dose, on the other hand, has been shown to improve Zn utilization in wheat in terms of higher grain yield and/or grain Zn-conc [29].

The Zn in wheat grain originates from two directions: the transfer process from post-anthesis canopy leaves to grains and the uptake process from soil by the roots [30]. Obviously, proper Zn application in soil can increase soil available Zn-conc, thereby enhancing the Zn translocation from soil to wheat plants [31]; Foliar Zn application mainly increases the transfer of Zn from canopy leaves to grains. The Zn translocation process in the soil–wheat system is regulated by the level of soil available Zn content. For instance, Liu et al. (2019) [30] found that grain Zn depended mainly on the uptake process from the soil when soil DTPA–Zn was greater than 7.15 mg kg^−1^, while the remobilization from the post-anthesis canopy leaves to the grains played as the main source when the soil Zn content was low.

Coastal soils often exhibit a higher content of salt and/or higher pH due to shallow groundwater tables, particularly for those in semi-arid and arid climates due to limited rainfall and high evaporation rates. Consequently, the high salt content present in saline soil could interfere with the Zn uptake process, resulting in a reduced Zn-acc in wheat plants [32]. Coastal saline land is an important resource for agricultural exploitation; taking the Yellow River Delta region in China as an example, screening better genotypes of crops and pastures tailored to the saline land has been formulated as a national strategy. To date, how coastal soil properties affect the soil Zn availability, and thereby the Zn translocation process in the soil–wheat systém, has not been fully understood. We hypothesized that Zn translocation from coastal soil to wheat was tightly associated with soil properties and negatively affected by soil salinity. The current study was thus conducted to test these hypotheses by examining the correlation of soil Zn supply level, soil properties and Zn translocation from coastal soil to wheat, as well as examining the effects of soil and foliar Zn application on Zn utilization of different wheat genotypes under coastal saline soils.

## 2. Results

### 2.1. Zn Translocation from Soil to Wheat Plant as Mediated by Soil Properties

Large variations were observed as anticipated for the measured soil properties parameters among 30 soil samples collected from different locations of the Yellow River Delta region (Table 1). PCA analysis using 16 measured soil chemical parameters showed that variations could be explained by 52.69% by the first two principal components, with PC1 and PC2 accounting for 33.57% and 19.12%, respectively (Supplementary Figure S1). PC1 mainly represented the ground nutritional status as manifested by concentrations of total N, total P, total Mg, total Ca, and total K. PC2 mainly represents the bioavailability of nutrients as manifested by NH_4_^+^-N, NO_3_^−^-N, Olsen-P, and the DTPA-Zn/Zn ratio (Supplementary Figure S1).

**Figure 1:**
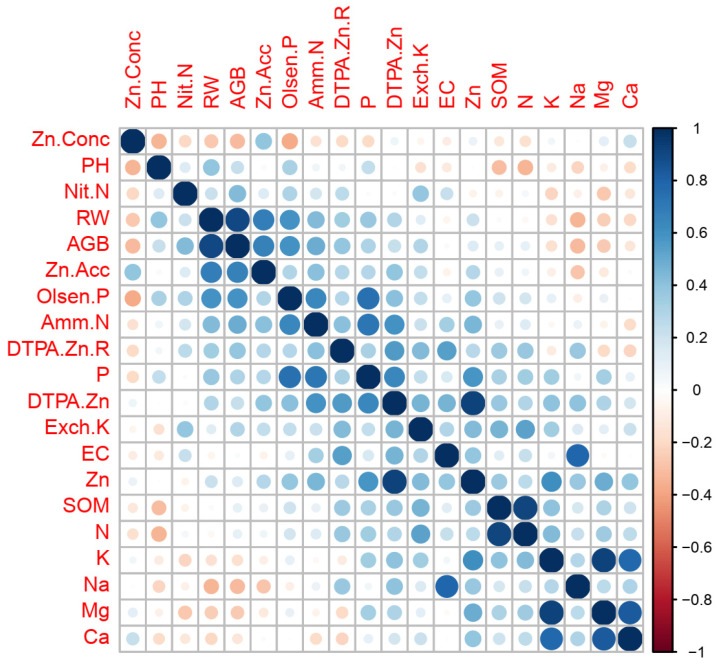
Correlations among the measured soil properties, aboveground biomass (AGB), root weight (RW), zinc concentration in the aboveground mass (Zn-Conc), and zinc accumulation (Zn-Acc). Note: The size of circle within each square indicates the correlation coefficient.

Correlation analysis (Figure 1) showed that the soil DTPA-Zn content positively correlated with soil total Zn concentration (r = 0.59, *p* < 0.01), EC (r = 0.47, *p* < 0.01), SOM (r = 0.38, *p* < 0.05), soil total P concentration (r = 0.64, *p* < 0.01), soil total K (r = 0.40, *p* < 0.05), NH_4_^+^-N (r = 0.60, *p* < 0.01), Olsen-P (r = 0.42, *p* < 0.05), and Exch-K (r = 0.47, *p* < 0.01). The available zinc to total zinc ratio positively correlated with soil EC (r = 0.49, *p* < 0.01) and negatively correlated with soil total Mg concentration (r = −0.42, *p* < 0.05).

Growth of wheat plants in terms of aboveground biomass and root dry weight measured at 8 weeks post-transplantation was significantly different among treatments using above 30 soils (*p* < 0.01). The aboveground biomass varied from 328.0 to 1729.0 mg/pot, while root dry weight varied from 49.0 to 365.0 mg/pot. Similarly, the Zn-conc of aboveground plants (*p* < 0.01) and total Zn-acc of aboveground plants (*p* < 0.01) were also significantly different. The Zn-conc of aboveground plants and total Zn-acc amount of aboveground plants were 24.8 to 116.6 mg kg^−1^ and 11.0 to 111.4 μg per pot, respectively.

**Figure 2:**
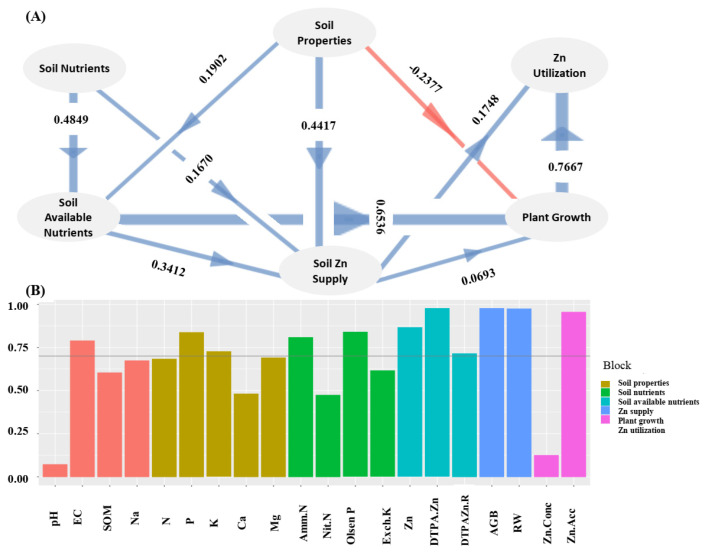
Inner model with path coefficients among six latent variables ‘soil properties’, ‘soil nutrients’, ‘soil Zn supply’, ‘plant growth’, and ‘Zn utilization’ in wheat (**A**) as revealed by PLS-PM. The loadings of the corresponding measured parameter for these six latent variables were shown (**B**).

The relationships among plant growth, Zn-acc, and soil properties were also revealed in correlation analysis (Figure 1). The Zn-conc in wheat seedling plants was negatively correlated with soil pH (r = −0.3365, *p* = 0.07) and Olsen-P (r = −0.3749, *p* < 0.05). AGB correlated positively with RW (r = 0.91, *p* < 0.01), and both AGB (r = −0.41, *p* < 0.05) and RW were negatively (r = −0.46, *p* < 0.01) correlated with soil Na concentration. The total Zn-acc in aboveground plants was positively correlated with soil DTPA-Zn content (r = 0.39, *p* < 0.05), Zn-conc in aboveground plants (r = 0.37, *p* < 0.05), AGB (r = 0.70, *p* < 0.01), and RW (r = 0.71, *p* < 0.01). Based on these correlations, it was assumed that Zn utilization in wheat could be affected by plant growth, soil Zn supply, soil properties, soil nutrients, and soil available nutrients. To test this hypothesis, a PLS-PM model with six latent variables, ‘plant growth’, ‘soil Zn supply’, ‘soil properties’, ‘soil nutrients’, ‘soil available nutrients’, and ‘Zn utilization’, was constructed. Indeed, soil Zn supply and plant growth had direct effects on Zn utilization in wheat as revealed in the established PLS-PM model (Figure 2). Soil properties, soil nutrients, and soil available nutrients had indirect effects on Zn utilization in wheat by affecting the soil Zn supply and/or plant growth.

### 2.2. The Effects of Zn Application on Wheat Zn Accumulation Mediated by Soil Salinity

**Figure 3:**
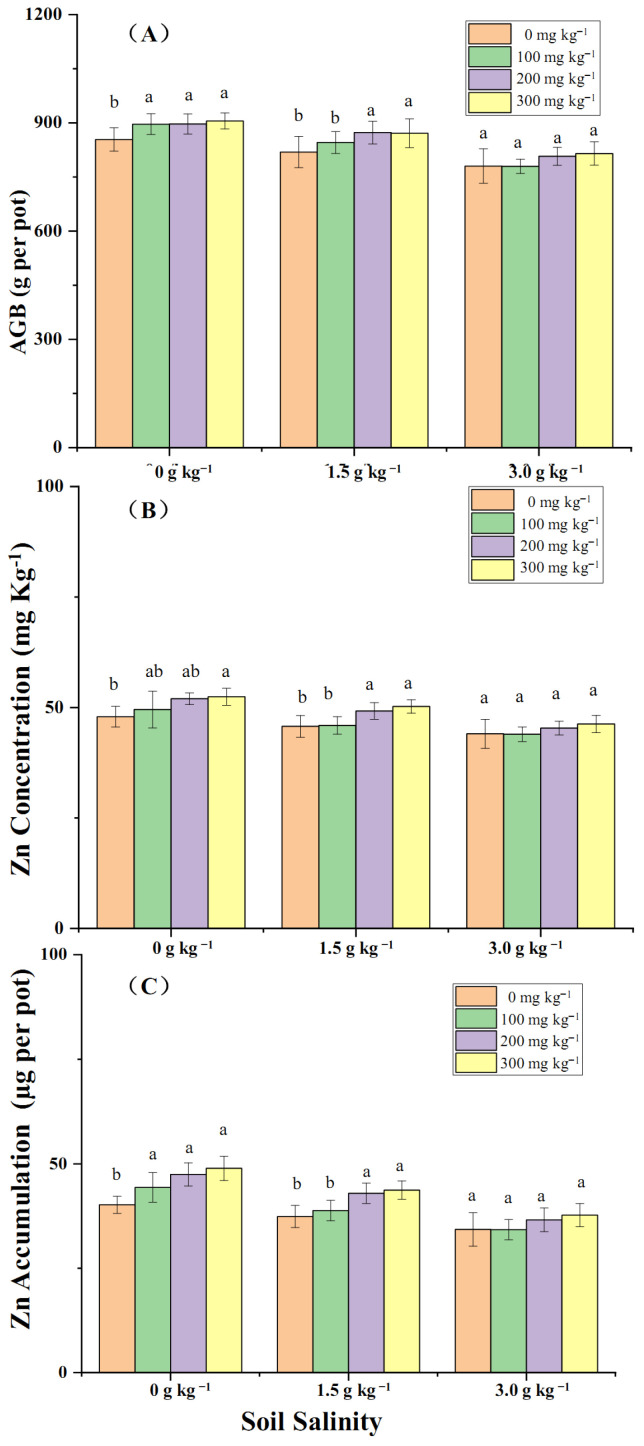
AGB (**A**), Zn-conc of aboveground plants (**B**), and Zn-acc in aboveground plants (**C**) of 8-week post-transplantation seedlings grown under three salinity and four Zn application levels. Note: different letters above the columns indicate a significant difference at 0.05 level.

Salinity significantly affected the aboveground biomass, Zn-conc of aboveground plants, and Zn-acc amount of aboveground plants measured at 8 weeks post-transplantation. Soil Zn application (100, 200, 300 mg kg^−1^) increased aboveground biomass and Zn-conc under no artificial imposed salinity (0 g kg^−1^ NaCl) and slight salinity treatments (1.5 g kg^−1^ NaCl) relative to no Zn application, but no further boosting effect was observed at a salinity of 3.0 g kg^−1^ NaCl (Figure 3A,B). Total Zn-acc was increased by 100, 200, and 300 mg kg^−1^ Zn application in conditions without artificial imposed salinity, while it was increased by a higher Zn application level (200, 300 mg kg^−1^) at a salinity of 1.5 g kg^−1^ NaCl. Zn application did not further increase the total Zn-acc in plants relative to no Zn application at a salinity of 3.0 g kg^−1^ NaCl.

**Figure 4:**
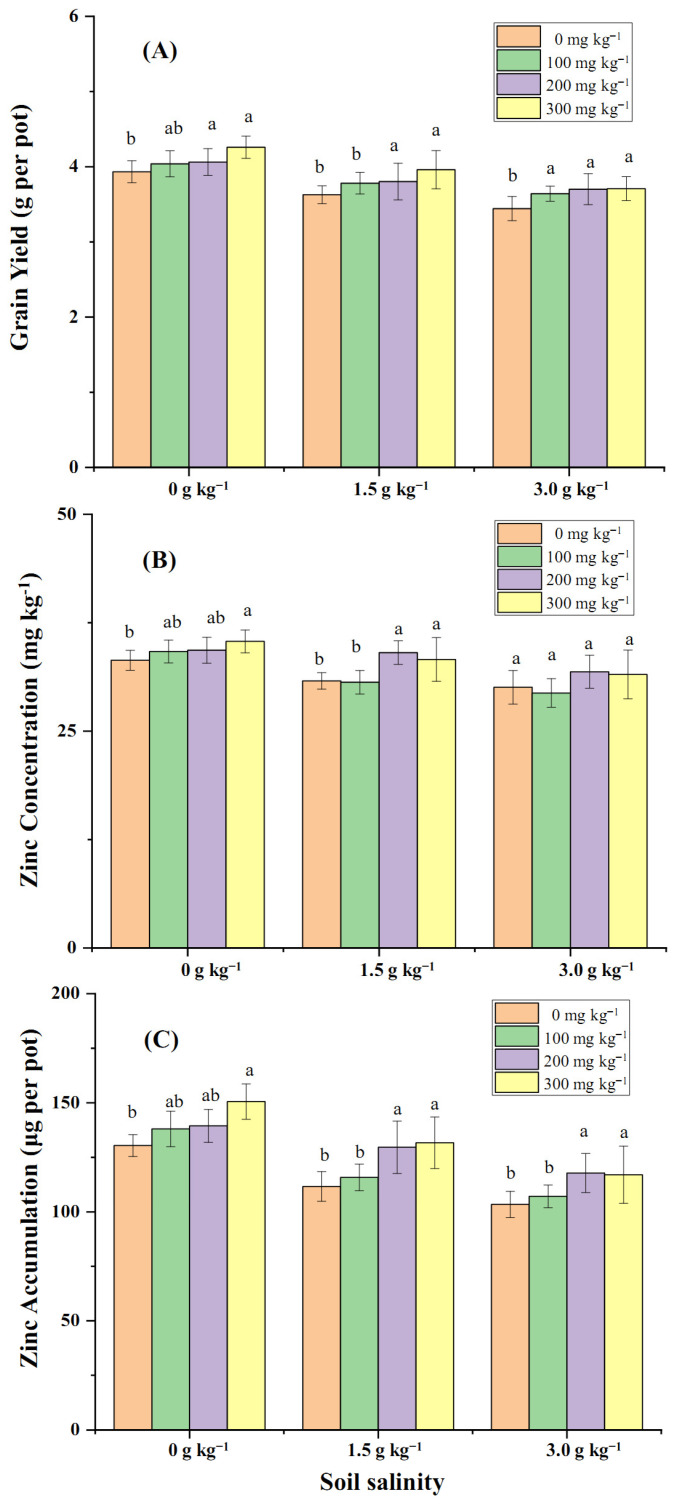
Grain yield (**A**), grain Zn-conc (**B**), and Zn-acc in grain (**C**) measured at maturity and grown under three salinity and four Zn application levels. Note: different letters above the columns indicate a significant difference at 0.05 level.

Grain yield, grain Zn-conc, and total Zn-acc in grain measured at maturity were significantly reduced by artificially imposed salinity (Figure 4). Soil Zn application (100, 200, 300 mg kg^−1^) increased grain yield relative to no Zn application at all three salinity levels (Figure 4A). Grain Zn-conc was increased by soil Zn application at salinities of 0 and 1.5 g kg^−1^ NaCl (Figure 4B), but no significant change at a salinity of 3.0 g kg^−1^ NaCl was observed. Total Zn-acc in grain was significantly increased by Zn application particularly at a dose of 200 and 300 mg kg^−1^ Zn application (Figure 4C).

### 2.3. Effect of Soil and Foliar Zn Application on Wheat Grain Yield and Grain Zn Concentration in Coastal Saline Fields

**Figure 5:**
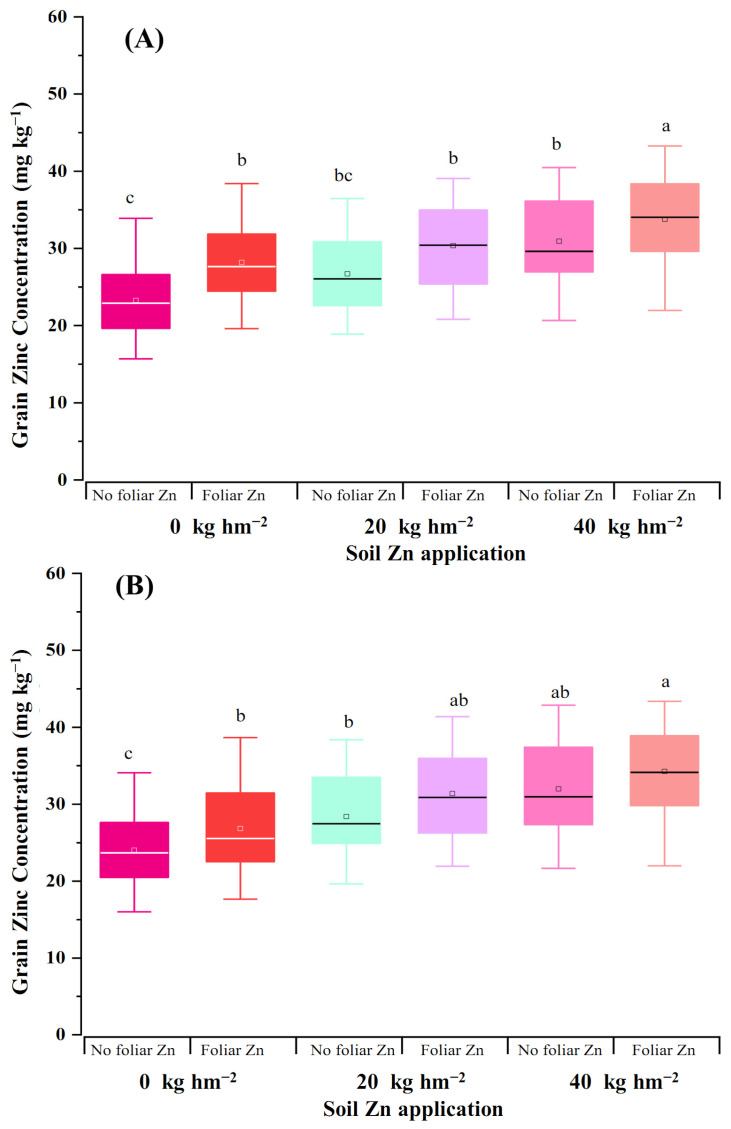
Grain Zn-conc of 20 wheat genotypes grown at three soil Zn levels and two foliar Zn levels under saline field conditions in the 2020–2021 (**A**) and 2021–2022 (**B**) seasons. Note: different letters above the columns indicate a significant difference at 0.05 level.

The effectiveness of soil and foliar Zn application on 20 wheat genotypes was further investigated on a field with low Zn availability. Soil Zn application significantly increased grain yield relative to the control treatment (0 kg hm^−2^ Zn) at both two growth seasons, with 40 kg/ha Zn application achieving the highest average grain yield, 5.36 t hm^−2^ in the 2020–2021 season and 5.53 t hm^−2^ in the 2021–2022 season (Supplementary Figure S2). However, within a given soil Zn level, foliar Zn application did not further increase grain yield (Supplementary Figure S3). Soil Zn application increased the average grain Zn-conc- of 20 wheat genotypes (Figure S4). By contrast, the effectiveness of foliar application was shown to be dependent on the dose of soil Zn application. Foliar Zn application increased grain Zn-conc significantly under the condition 0 kg hm^−2^ soil Zn application but not for conditions under 20 kg hm^−2^ or 40 kg hm^−2^ soil Zn application (Figure 5). On the other hand, a significant genotypic difference was also observed in terms of grain yield and grain Zn-conc (Supplementary Table S2).

## 3. Discussion

### 3.1. Zn Translocation from Soil to Wheat Mediated by Soil Properties

Soil Zn availability is closely associated with the soil properties (pH, organic matter content, soil nitrogen, phosphorus content, etc.) [32,33]. The formation of aqueous Zn-chloride complexes in saline conditions has been demonstrated to reduce the availability of free Zn^2+^ ions, thereby diminishing the efficiency of Zn application in salt-affected conditions [34]. The salt component in the Yellow River Delta is mainly NaCl. Therefore, the reduced Zn-acc in the wheat plant in the current study could be attributed partly to the decreased Zn availability in the soil. The releasing capability of nutrients from soil to solution can be estimated by measuring the soil available concentration of nutrients such as N, P, K, and Zn. The DTPA-Zn content positively correlated with ammonia-N, Olsen-P, and exchangeable K in our study, suggesting that a soil with higher availability of N, P, and K has a tendency to have more Zn available. The soil Zn content positively correlated with available P when the available P concentration did not exceed 150 mg kg^−1^ [35]. Consistently, the DTPA-Zn content positively correlated with the Olsen-P concentration, which varied from 24.90 mg kg^−1^ to 60.40 mg kg^−1^ in our current study.

Soil organic matter and pH were two indexes among the investigated soil properties that correlated best with the distribution of Zn forms in soils [36]. The correlation between soil DTPA-Zn and organic matter also varies with the change in SOM content. An increase in organic matter concentration to a certain extent could increase the soil Zn content [20]. However, an increase in SOM exceeding 90 g kg^−1^ resulted in a reduction in Zn content [35]. SOM, which varied from 3.70 to 36.10 g kg^−1^ in the current study, as anticipated, was positively correlated with DTPA-Zn content. The correlation between pH and soil available Zn content in the current study was non-significant and weak; this might be due to the narrow pH range (6.25–8.35) of the soils used in this study. The Zn availability at higher pH decreases due to lower solubility of minerals containing Zn and increased Zn binding to negatively charged clay particles and Fe and Mn-oxides. Soil properties indexes such as soil clay content and Fe and Mn-oxides content should, therefore, also be taken into account while evaluating the influencing factors associated with Zn availability.

The interactions between soil factors and wheat plants are complex and may vary depending on the soil properties and wheat genotype. The difference in soil properties could thereby partially contribute to the variation in grain Zn-conc in crops grown in different locations [37,38,39]. As anticipated, the Zn-conc in wheat plants grown under different soils varied largely in the current study. Higher pH often leads to a decrease in Zn availability [3,12]. Consistently, Zn-conc in wheat seedling plants in the current study was negatively correlated with soil pH, suggesting a reduced availability to plants caused by a higher pH. Moreover, Zn-conc in wheat seedling plants showed a negative correlation with soil Olsen-P concentration increases, which was in agreement with [40], in which reduced soil available P was associated with higher grain Zn-conc.

Zn-acc in plants depends on the capability to assimilate the uptaken Zn into dry matter. Total Zn-acc amount in plants correlates positively with aboveground biomass in this current study, suggesting a better growth performance promoted by Zn uptake. Moreover, total Zn-acc in aboveground plants was positively correlated with soil DTPA-Zn concentration, suggesting soil Zn supply levels had a significant effect on Zn-acc, consistent with Recena et al. (2021), which showed that the available Zn is relevant in explaining Zn uptake by plants [41]. On the contrary, no relationship was found between Zn uptake by plants and the DTPA-Zn in the research of Moreno-Lora and Delgado (2020) [42]. The disagreement might be due to the fact that the soil DTPA-Zn content in our study had a larger span and a higher average.

Clarifying the relationship among soil properties, Zn availability, and Zn utilization would be helpful for making suitable measures to improve Zn translocation from soil to plant. The established PLS-PM model supported that soil Zn supply and plant growth had direct effects on Zn utilization in wheat, while soil properties, soil nutrients, and soil available nutrients had indirect effects on Zn utilization in wheat by affecting soil Zn supply and/or plant growth. This model suggested that soil Zn supply level and plant growth were two factors directly related to Zn utilization, while soil properties, soil nutrients, and soil available nutrients were indirectly related.

### 3.2. Effectiveness of Zn Application on Zn Translocation from Soil to Wheat Mediated by Salt Stress

Insufficient Zn supply resulted in reduced water usage, delayed head emergence, and the depressed grain yield of Zn-inefficient wheat genotype [43]. The application of Zn fertilizer to soils with low Zn supply levels is therefore a key approach to support plant normal growth and satisfactory grain yield. Proper soil Zn application could enhance Zn translocation from soil to plant; however, the effectiveness of soil Zn application was dependent on the soil’s natural Zn level. Zn fertilizer explained most of the Zn uptake by crops in Zn-deficient soils; Zn application may not significantly affect grain yield under soils with sufficient Zn [41]. Soil Zn application at higher doses (200, 300 mg kg^−1^) increased grain yield at all three salinity levels in the current pot study, suggesting that the Zn availability of the original soils combined with salinity was insufficient. Grain Zn-concen was increased significantly by imposing a dose of 300 mg kg^−1^ Zn at a salinity level of 0 g kg^−1^ and 1.5 g kg^−1^.

Similarly, the effectiveness of foliar Zn application on grain yield and grain Zn-conc was also dependent on the soil natural Zn level. Foliar did not increase grain yield while it significantly improved the grain Zn-conc- of wheat by 28% and 89% in Zn non-deficient soils [44]. Foliar Zn application did not further increase grain yield but increased the average grain Zn-conc of 20 wheat genotypes in our current study.

On average, the grain Zn-conc of 20 wheat genotypes involved in this study was below the Zn biofortification target. A large genotypic difference was observed for grain Zn-conc, with five genotypes, “Line 1280”, “Zhengmai 9405”, “Big grain No. 1”, “Taishan 4033”, and “Line 1051”, exceeding the Zn biofortification target of 40 mg kg^−1^ under soil and/or foliar treatments. Selecting genotypes with higher Zn-acc and optimizing with a proper Zn management are therefore required to reach the Zn biofortification of wheat grown under coastal soils.

## 4. Materials and Methods

### 4.1. Pot Study Using Soils from Different Locations

In order to examine the effects of soil properties on Zn translocation from soil to wheat, top soils (0–20 cm) were sampled from 30 different locations of the coastal saline region of the Yellow River Delta (Supplementary Table S1). The sampled soils were then used as the culture substrate in a greenhouse pot study at Shandong University of Aeronautics.

A completely random block design experiment with one factor ‘soil location’ was conducted from October 2020 to June 2021. Wheat (*Triticum aestivum* L.) cv. LX99, a control variety in North China, was used as the experimental cultivar. In total, 30 different soils were regarded as different levels of the factor ‘soil location’. Seed germination was initiated in the dark at room temperature for 3 days; three uniform seedlings were subsequently transplanted into a plastic pot (height, 18.0 cm; diameter, 16.0 cm) filled with 2.0 kg of soil. Three pots (replications) were assigned for each soil sample. The light and temperature conditions were set to 16 h light at 25 ± 3 °C and 8 h dark at 18 ± 3 °C, photosynthetic photon flux density set at 120 μmol m^−2^ s^−1^, and relative humidity maintained at 50–60%; the relative water content in the soil was maintained around 70%. The plant growth difference among these 30 different soils was assumed to be caused by differences in soil properties. Plants in this pot study were harvested at 8 weeks post-transplantation.

### 4.2. Pot Study Examining the Effects of Zn Application and Salinity

A completely random block pot study with two factors, “salinity” and “Zn supply”, was applied. A soil sample collected from a farmland (37°13′27.00″ N 118°01′30.00″ E) in Bin Cheng district, Bin Zhou, Shan Dong province, China, was used as the culture substrate. The original soil had a pH of 7.8, total N of 1.0 g/kg, NaHCO_3_-extractable phosphorus (Olsen-P) of 31.8 mg kg^−1^, total Zn of 60.8 mg kg^−1^, and DTPA-Zn of 3.5 mg kg^−1^. After mixing well, a 2.0 kg soil sample was filled into a plastic pot (height, 18.0 cm; diameter, 16.0 cm). Three salinity levels (NaCl added into soil as 0 g, 1.5 g, and 3.0 g per 1 kg soil) were imposed on corresponding pots by watering the required amount of NaCl solution or distilled water for control pots. The salt-stress-imposed pots were allowed to stay in greenhouse conditions for two months. Then, four Zn application levels (Zn added into soil as 0 mg, 100 mg, 200 mg, and 300 mg per 1 kg soil by applying required amount of ZnSO_4_, i.e., 246.9 mg, 493.8 mg, and 740.7 mg per 1 kg soil, respectively) were applied by mixing the required amount of ZnSO_4_ with the salt-stress imposed soil. Wheat (*Triticum aestivum* L.) cv. LX99 was used as the experimental material. The growth condition was controlled in the same way as described above in Section 2.1. Plants in this pot study were harvested at two stages, one at 8 weeks post-transplantation and another at maturity.

### 4.3. Field Trial

Field trials were conducted during the 2020–2021 and 2021–2022 wheat growing seasons in a coastal saline field at the western line of Bohai Gulf within Wudi, Shandong Province, China (37°56′24″ N 117°49′48″ E). This experimental site experiences semi-drought conditions with pronounced seasonal fluctuations due to its temperate continental monsoon climate. The initial soil characteristics of the three saline fields were as follows: pH (8.1), EC (6.3 dS m^−1^), total N (0.9 g kg^−1^), Olsen-P (8.4 mg kg^−1^), total Zn (17.8 mg kg^−1^), and DTPA-Zn (0.9 mg kg^−1^).

A split-split-plot design was applied. Three soil Zn application levels, 0 kg hm^−2^, 20 kg hm^−2^, and 40 kg hm^−2^ Zn, were arranged as three main plots by applying ZnSO_4_ in the required amount into the soil of each treatment. Two foliar Zn application levels, no foliar application, and foliar application were set as two sub-plots. Twenty wheat genotypes were regarded as sub-sub-plots and planted with three replications.

Each main plot received a specific Zn supply level and equal amounts of N (200 kg hm^−2^) applied in the form of Urea, P (150 kg hm^−2^) applied in the form of Ca(H_2_PO_4_)_2_, and K (120 kg hm^−2^) applied in the form of KCl. All three chemicals were purchased from Xilong Scientific Co., Ltd. Chengdu, China. For both growth seasons, foliar Zn application was applied at the heading stage by spraying 15 kg hm^−2^. Zn in the form of ZnSO_4_. 250 seeds m^−2^ were manually sown into each sub-sub-plot (1 m × 2 m) in mid-October in both two growth seasons. Mature plants from each plot were harvested at mid-June each season.

### 4.4. Measurement of Soil Chemical Indexes

pH and electrical conductivity were measured using a soil extraction solution by a pH meter and a conductivity meter (Shanghai Leici Instrument Co., Ltd., Shanghai, China), respectively. The extraction solution was collected by allowing a mixture of air-dried soil and water (g:mL, 1:5) to be homogenized for 5 min and subsequently passing through a vacuum pump at −0.08 MPa (ShaoXing Supo Instrument Co., Ltd., ShaoXing, China). Soil organic matter (SOM) content was determined using the K_2_Cr_2_O_7_ method, and the absorbance value was measured at a wavelength of 600 nm using a UV–Visible spectrophotometer (TU-1901, Beijing Purkinje General Instrument Co., Ltd., Beijing, China). The content of soil organic matter was converted from Total Organic Carbon (TOC), SOM (%) = TOC (%) × 1.724. The content of soil total nitrogen was determined using the Kjeldahl method after digestion by sulfuric acid. Ammonia-N (NH_4_^+^-N) and nitrate-N (NO_3_^−^-N) were measured by the colorimetric method after extraction by 1 M KCl. The soil available P content (Olsen-P) was measured by the molybdenum antimony colorimetric method using 0.5 M NaHCO_3_ extraction solution.

### 4.5. Elemental Measurement of Soil and Plants

Oven-dried plant samples were ground to a fine powder, then a portion of the sample (50 mg) was digested in a mixture of 15 mL HNO_3_ and 2 mL H_2_O_2_ at 150 °C for 1 h, by using an automatic microwave digester (CEM MARS 6™ Microwave Digestion System, Matthews, NC, USA). To determine the content of total Na, K, Ca, Mg, P, and Zn in soil, soil samples were firstly air-dried at room temperature, then homogenized by grounding into fine powder; subsequently, a portion of soil sample (100) mg from each sampling site or treatment was digested in a mixture of 9 mL HNO_3_, 3 mL HCl, 3 mL HF, and 2 mL H_2_O_2_ at 150 °C for 1 h. Concentrations of Na, K, Ca, Mg, P, and Zn in plant and soil were determined by Inductively Coupled Plasma—Optical Emission Spectrometer (ICP–OES) (Thermo Fisher Scientific, Waltham, MA, USA). The fraction of soil exchangeable K was obtained by extracting air-dried soil with ammonium acetate solution. The fraction of soil DTPA-Zn was extracted using extraction buffer (0.005 M DTPA, 0.01 M CaCl_2_, 0.1 M TEA, pH 7.3) and subsequently measured by ICP–OES.

### 4.6. Statistical Analysis

ANOVA was conducted to examine the effect of the tested factor using the SPSS 16.0 package (IBM, New York, NY, USA); significance levels (α) were set as 0.05 and 0.01, representing significantly and very significantly different, respectively. Pearson’s correlation analysis and PCA analysis were conducted using Origin 2018 (OriginLab Co., Northampton, MA, USA) to examine relationships among measured variables. Based on correlation and PCA analysis, the measured soil chemical parameters, plant growth parameters, and Zn-acc parameters were classified into six latent variables: ‘soil properties’, ‘soil nutrients’, ‘soil available nutrients’, ‘plant growth’, ‘soil Zn supply’ and ‘Zn utilization’. Partial Least Squares Path Modeling (PLS-PM) was applied to establish the relationships among these latent variables using the plspm package in R.

## 5. Conclusions

Firstly, this study explored the correlations among soil properties, Zn availability, and Zn utilization in wheat. Higher soil pH and/or Olsen-P reduced Zn-conc in wheat plants, while higher soil EC value reduced both aboveground biomass and root weight. Zn utilization in wheat grown under coastal soils can be affected directly by soil Zn supply and plant growth, while indirectly affected by soil properties, soil nutrients, and soil available nutrients. Secondly, the effectiveness of the Zn application on grain yield and/or grain Zn-conc in wheat was shown to be dependent on the soil natural Zn level and soil salinity as revealed by a pot study and field trial. Grain yield and grain Zn-conc were increased by Zn application under soils with a low salinity, while grain Zn-conc was not increased by Zn application under soils with high salinity. Thirdly, field trials suggested that soil Zn application (20–40 kg hm^−2^) could increase grain Zn-conc under soils with low Zn availability. Moreover, a large genotypic difference was observed among 20 wheat genotypes under coastal saline soils. Adjusting the Zn application tailored to suitable wheat genotypes according to soil properties is promising to reach the Zn biofortification target and a satisfactory yield for wheat grown under coastal saline soils.

## Figures and Tables

**Table 1:** Measured soil properties parameters of 30 soil samples.

Measured Soil Chemical Parameters	Average ± SD	Minimum Value	Median Value
pH	7.32 ± 0.64	6.25	7.49
EC (dS m^−1^)	5.76 ± 5.17	1.02	3.42
SOM (g kg^−1^)	12.73 ± 6.86	3.70	12.40
Na (g kg^−1^)	0.52 ± 0.32	0.23	0.41
N (g kg^−1^)	0.75 ± 0.40	0.10	0.72
P (g kg^−1^)	0.84 ± 0.35	0.48	0.75
K (g kg^−1^)	2.37 ± 0.91	0.96	2.24
Ca (g kg^−1^)	32.80 ± 5.42	24.36	30.82
Mg (g kg^−1^)	8.60 ± 1.72	5.70	8.56
NH_4_^+^-N (mg kg^−1^)	120.00 ± 12.00	20.00	100.00
NO_3_^−^-N (mg kg^−1^)	53.49 ± 80.49	12.34	35.00
Olsen-P (mg kg^−1^)	40.46 ± 11.35	24.90	36.20
Exch-K(mg kg^−1^)	192.06 ± 71.88	68.64	186.75
Zn (mg kg^−1^)	58.14 ± 57.52	14.60	44.25
DTPA-Zn (mg kg^−1^)	3.29 ± 5.65	0.60	1.60
DTPA/Zn R (%)	4.88 ± 2.97	1.34	3.55

Abbreviations: soil reaction (pH), electrical conductivity (EC), soil organic matter (SOM), total sodium (Na), total nitrogen (N), total phosphorus (P), total potassium (K), total calcium (Ca), total magnesium (Mg), ammonia nitrogen (Amm.N; NH_4_^+^-N), nitrate nitrogen (Nit.N; NO_3_^−^-N), available phosphorus (Olsen-P), exchangeable potassium (Exch-K), total zinc (Zn), available Zn (DTPA-Zn), available zinc to total zinc ratio (DTPA/Zn R).

## Data Availability

The original contributions presented in the study are included in the article/Supplementary Materials; further inquiries can be directed to the corresponding author.

## Supplementary Materials

The following supporting information can be downloaded at https://www.mdpi.com/article/10.3390/plants14131971/s1. Figure S1: Principal component analysis of 16 measured soil chemical parameters; Figure S2: Grain yield of 20 wheat genotypes grown at three soil zinc levels (0 kg hm^−2^, 20 kg hm^−2^, and 40 kg hm^−2^ Zn by applying ZnSO_4_) under saline field conditions in the 2020–2021 (A) and 2021–2022 (B) seasons; Figure S3: Average grain yield of 20 wheat genotypes grown at three soil zinc levels (0 kg hm^−2^, 20 kg hm^−2^, and 40 kg hm^−2^ Zn by applying ZnSO_4_) and two foliar zinc levels (no foliar application and foliar application) under saline field conditions in 2020–2021 (A) and 2021–2022 (B) seasons; Figure S4: Grain zinc concentration of 20 wheat genotypes grown at three soil zinc levels (0 kg hm^−2^, 20 kg hm^−2^, and 40 kg hm^−2^ Zn by applying ZnSO_4_) under saline field conditions in the 2020–2021 (A) and 2021–2022 (B) seasons; Table S1: Latitude and longitude coordinates of sampling sites; Table S2: Grain yield and grain zinc concentration of 20 wheat genotypes.

## References

[1] Stangoulis J.C.R., Knez M. (2022). Biofortification of major crop plants with iron and zinc-achievements and future directions. Plant Soil..

[2] Liu H., Wang Z., Li F., Li K., Yang N., Yang Y., Huang D., Liang D., Zhao H., Mao H. (2014). Grain iron and zinc concentrations of wheat and their relationships to yield in major wheat production areas in China. Field Crop. Res..

[3] Cakmak I., Kutman U.B. (2018). Agronomic biofortifcation of cereals with zinc: A review. Eur. J. Soil Sci..

[4] Roy C., Kumar S., Ranjan R.D., Kumhar S.R., Govindan V. (2022). Genomic approaches for improving grain zinc and iron content in wheat. Front. Genet..

[5] Guo Z., Zhang X., Wang L., Wang X., Wang R., Hui X., Wang S., Wang Z., Shi M. (2021). Selecting high zinc wheat cultivars increases grain zinc bioavailability. J. Agric. Food Chem..

[6] Senguttuvel P., Padmavathi G., Jasmine C., Sanjeeva Rao D., Neeraja C.N., Jaldhani V., Beulah P., Gobinath R., Aravind Kumar J., Sai Prasad S.V. (2023). Rice biofortification: Breeding and genomic approaches for genetic enhancement of grain zinc and iron contents. Front. Plant Sci..

[7] Yang M., Li Y., Liu Z., Tian J., Liang L., Qiu Y., Wang G., Du Q., Cheng D., Cai H. (2020). A high activity zinc transporter oszip9 mediates zinc uptake in rice. Plant J..

[8] Ning M., Liu S.J., Deng F., Huang L., Li H., Che J., Yamaji N., Hu F., Lei G.J. (2023). A vacuolar transporter plays important roles in zinc and cadmium accumulation in rice grain. New Phytol..

[9] Kamran A., Ghazanfar M., Khan J.S., Pervaiz S., Siddiqui M.H., Alamri S. (2023). Zinc Absorption through Leaves and Subsequent Translocation to the Grains of Bread Wheat after Foliar Spray. Agriculture.

[10] Khalid S., Amanullah, Ahmed I. (2022). Enhancing Zinc Biofortification of Wheat through Integration of Zinc, Compost, and Zinc-Solubilizing Bacteria. Agriculture.

[11] Alharbi K., Hafez E.M., Omara A.E.D., Rashwan E., Alshaal T. (2023). Zinc oxide nanoparticles and PGPR strengthen salinity tolerance and productivity of wheat irrigated with saline water in sodic-saline soil. Plant Soil..

[12] Alloway B.J. (2009). Soil factors associated with zinc deficiency in crops and humans. Environ. Geochem. HLTH.

[13] Palmer B., Guppy C., Nachimuthu G., Hulugalle N. (2023). Changes in micronutrient concentrations under minimum tillage and cotton-based crop rotations in irrigated vertisols. Soil Till. Res..

[14] Mehlich A. (1984). Mehlich No. 3 soil test extractant: A modification of Mehlich No. 2. Commun. Soil Sci. Plant Anal..

[15] Hartemink A.E., Barrow N.J. (2023). Soil pH-nutrient relationships: The diagram. Plant Soil..

[16] Barrow N.J., Hartemink A.E. (2023). The efects of pH on nutrient availability depend on both soils and plants. Plant Soil..

[17] Van Eynde E., Groenenberg J.E., Hofand E., Comans R.N.J. (2022). Solid-solution partitioning of micronutrients Zn, Cu and B in tropical soils: Mechanistic and empirical models. Geoderma.

[18] Hernandez-Soriano M.C., Peña A., Mingorance M.D. (2013). Soluble metal pool as afected by soil addition with organic inputs. Environ. Toxicol. Chem..

[19] Tella M., Bravin M.N., Thuriès L., Cazevieille P., Chevassus-Rosset C., Collin B., Chaurand P., Legros S., Doelsch E. (2016). Increased zinc and copper availability in organic waste amended soil potentially involving distinct release mechanisms. Environ. Pollut..

[20] Soltani S., Khoshgoftarmanesh A.H., Afyuni M., Shrivani M., Schulin R. (2014). The effect of preceding crop on wheat grain zinc concentration and its relationship to total amino acids and dissolved organic carbon in rhizosphere soil solution. Biol. Fert. Soils.

[21] Laurent C., Bravin M.N., Crouzet O., Lamy I. (2024). Does a decade of soil organic fertilization promote copper and zinc phytoavailability? Evidence from a laboratory biotest with field-collected soil samples. Sci. Total Environ..

[22] Dawar K., Ali W., Bibi H., Mian I.A., Ahmad M.A., Hussain M.B., Ali M., Ali S., Fahad S., Rehman S.u. (2022). Effect of Different Levels of Zinc and Compost on Yield and Yield Components of Wheat. Agronomy.

[23] Van Eynde E., Fendrich A.N., Ballabio C., Panagos P. (2023). Spatial assessment of topsoil zinc concentrations in Europe. Sci. Total Environ..

[24] He H., Wu M., Su R., Zhang K., Chang C., Peng Q., Dong Z., Pang J., Lambers H. (2021). Strong phosphorus (P)-zinc (Zn) interactions in a calcareous soil-alfalfa system suggest that rational P fertilization should be considered for Zn biofortification on Zn-deficient soils and phytoremediation of Zn-contaminated soils. Plant Soil.

[25] Watts-Williams S.J., Smith F.A., McLaughlin M.J., Patti A.F., Cavagnaro T.R. (2015). How important is the mycorrhizal pathway for plant Zn uptake?. Plant Soil.

[26] Zhang W., Chen X., Liu Y., Liu D., Chen X., Zou C. (2017). Zinc uptake by roots and accumulation in maize plants as affected by phosphorus application and arbuscular mycorrhizal colonization. Plant Soil.

[27] Coccina A., Cavagnaro T.R., Pellegrino E., Ercoli L., McLaughlin M.J., Watts-Williams S.J. (2019). The mycorrhizal pathway of zinc uptake contributes to zinc accumulation in barley and wheat grain. BMC Plant Biol..

[28] Ding J., Liu L., Wang C., Shi L., Xu F., Cai H. (2021). High level of zinc triggers phosphorus starvation by inhibiting root-to-shoot translocation and preferential distribution of phosphorus in rice plants. Environ. Pollut..

[29] Li M., Yang X.W., Tian X.H., Wang S.X., Chen Y.L. (2014). Effect of Nitrogen Fertilizer and Foliar Zinc Application at Different Growth Stages on Zinc Translocation and Utilization Efficiency in Winter Wheat. Cereal Res. Commun..

[30] Liu D.Y., Liu Y.M., Zhang W., Chen X.P., Zou C.Q. (2019). Zinc Uptake, Translocation, and Remobilization in Winter Wheat as Affected by Soil Application of Zn Fertilizer. Front. Plant Sci..

[31] Liu Y.M., Liu D.Y., Zhao Q.Y., Zhang W., Chen X.X., Xu S.J., Zou C.Q. (2020). Zinc fractions in soils and uptake in winter wheat as affected by repeated applications of zinc fertilizer. Soil Till. Res..

[32] Zhao D.Y., Zhang Z.W., Yuan Y.R., Zhang X.L., Zhao W.F., Li X.P., Wang J., Siddique K.H.M. (2022). Accumulation of zinc, iron and selenium in wheat as affected by phosphorus supply in salinised condition. Crop Pasture Sci..

[33] Guo Z.K., Wang X.S., Zhang X.M., Wang R.Z., Wang S., Chen Y.L., Liu J.S., Tian H., Wang Z.H., Shi M. (2023). Rhizosphere microbiome-related changes in soil zinc and phosphorus availability improve grain zinc concentration of wheat. Plant Soil.

[34] Hanif M.D., Bullen J.C., Plancherel Y., Kirby M., Kirk G.J.D., Weiß D.J. (2024). Significant effect of salinity on zinc adsorption on tropical coastal and floodplain soils. Eur. J. Soil Sci..

[35] Jiang Y., Gao W.W., Zhao J.L., Chen Q., Liang D., Xu C., Huang L.S., Ruan L.M. (2018). Analysis of influencing factors on soil Zn content using generalized additive model. Sci. Rep..

[36] Obrador A., Alvarez J.M., Lopez-Valdivia L.M., Gonzalez D., Novillo J., Rico M.I. (2007). Relationships of soil properties with Mn and Zn distribution in acidic soils and their uptake by a barley crop. Geoderma.

[37] Li C., Guo Z., Wang X., Ma Y., Liu J., Shi M., Zhang D., Malhi S.S., Siddique K.H.M., Wang Z.H. (2023). Field-scale studies quantify limitations for wheat grain zinc biofortification in dryland areas. Eur. J. Agron..

[38] Shen Y.T., Wiita E., Nghiem A.A., Liu J.Y., Haque E., Austin R.N., Seng C.Y., Phan K., Zheng Y., Bostick B.C. (2023). Zinc localization and speciation in rice grain under variable soil zinc deficiency. Plant Soil.

[39] Van Eynde E., Breure M.S., Chikowo R., Njoroge S., Comans R.N.J., Hoffland E. (2023). Soil zinc fertilisation does not increase maize yields in 17 out of 19 sites in Sub-Saharan Africa but improves nutritional maize quality in most sites. Plant Soil.

[40] Hui X., Wang X., Luo L., Wang S., Guo Z., Shi M., Wang R., Lyons G., Chen Y., Cakmak I. (2022). Wheat grain zinc concentration as affected by soil nitrogen and phosphorus availability and root mycorrhizal colonization. Eur. J. Agron..

[41] Recena R., García-López A.M., Delgado A. (2021). Zinc Uptake by Plants as Affected by Fertilization with Zn Sulfate, Phosphorus Availability, and Soil Properties. Agronomy.

[42] Moreno-Lora A., Delgado A. (2020). Factors determining Zn availability and uptake by plants in soils developed under Mediterranean climate. Geoderma.

[43] Nable R.O., Webb M.J. (1993). Further evidence that zinc is required throughout the root zone for optimal plant growth and development. Plant Soil.

[44] Wang J., Mao H., Zhao H., Huang D., Wang Z. (2012). Different increases in maize and wheat grain zinc concentrations caused by soil and foliar applications of zinc in Loess Plateau, China. Field Crop. Res..

