# Supplementary material for: Zinc Translocation from Coastal Soil to Wheat as Mediated by Zinc Supply Levels and Soil Properties
Source: Plants (Basel). 2025 Jun 27;14(13):1971. doi: 10.3390/plants14131971 (PMC12251930; doi:10.3390/plants14131971)
Supplement: Supplementary file 1 [file plants-14-01971-s001.zip › plants-3699129-supplementary.pdf]

Zinc translocation from coastal soil to wheat as mediated by zinc supply levels and soil properties

Deyong Zhao[1,2,3]*, Jie Dong [1], Yan Li [1]

1 College of Biological and Pharmaceutical Engineering, Shandong University of Aeronautics, Binzhou, Shandong 256603, P.R. China
2 Shandong Key Laboratory of Eco-Environmental Science for Yellow River Delta, Shandong University of Aeronautics, Binzhou, Shandong 256603, P.R. China
3 Shandong Engineering and Technology Research Center for Fragile Ecological Belt of Yellow River Delta, Binzhou, Shandong 256603, P.R. China
*Correspondence: dyzhao@sdua.edu.cn (D.Z.)

Supplementary figures and tables

Supplementary Table S1. Latitude and Longitude Coordinates of Sampling Sites

Sample No	Latitude and Longitude Coordinates	
	North Latitude （N)	East Longitude（E）
1	37°29′28〃	118°05′09〃
2	37°30′03〃	118°08′29〃
3	37°35′44〃	118°18′47〃
4	37°41′02〃	118°27′21〃
5	37°47′10〃	118°33′35〃
6	37°47′10〃	118°33′33〃
7	37°47′43〃	118°35′16〃
8	37°49′27〃	118°35′18〃
9	37°51′28〃	118°35′48〃
10	37°53′35〃	118°42′04〃
11	37°54′25〃	118°46′41〃
12	37°56′18〃	118°49′05〃
13	37°53′41〃	118°28′32〃
14	37°52′46〃	118°28′20〃
15	37°51′04〃	118°28′27〃
16	37°50′51〃	118°26′02〃
17	37°50′22〃	118°22′13〃
18	37°50′35〃	118°19′55〃
19	37°50′33〃	118°19′56〃
20	37°48′39〃	118°15′12〃
21	37°46′16〃	118°11′38〃
22	37°46′18〃	118°11′37〃
23	37°43′39〃	118°08′30〃
24	37°38′21〃	118°06′10〃
25	37°37′25〃	118°05′50〃
26	37°37′27〃	118°05′50〃
27	37°35′22〃	118°05′44〃
28	37°34′05〃	118°04′52〃
29	37°34′05〃	118°04′50〃
30	37°28′38〃	118°01′26〃

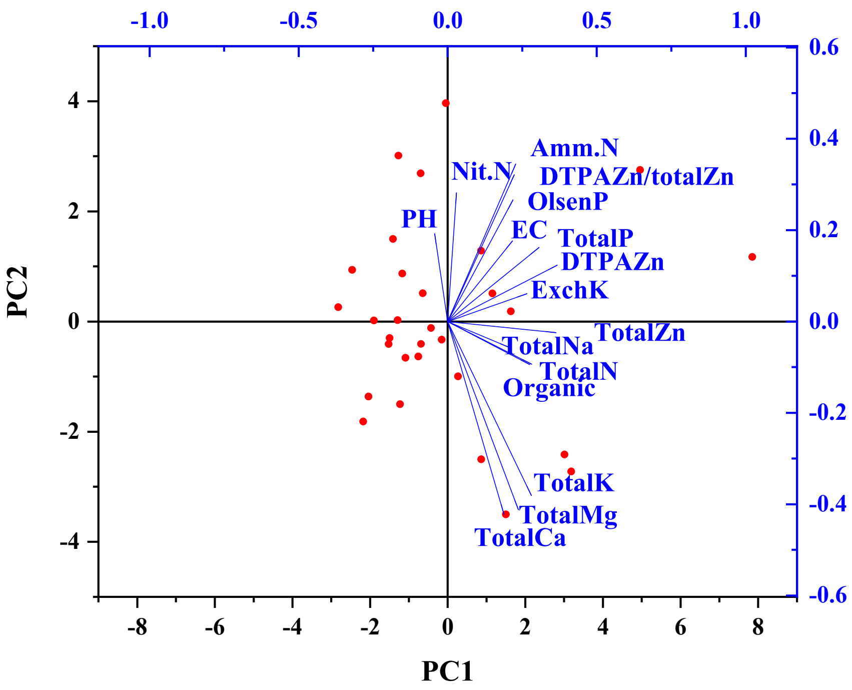

Fig S1. Principal component analysis of 16 measured soil chemical parameters.

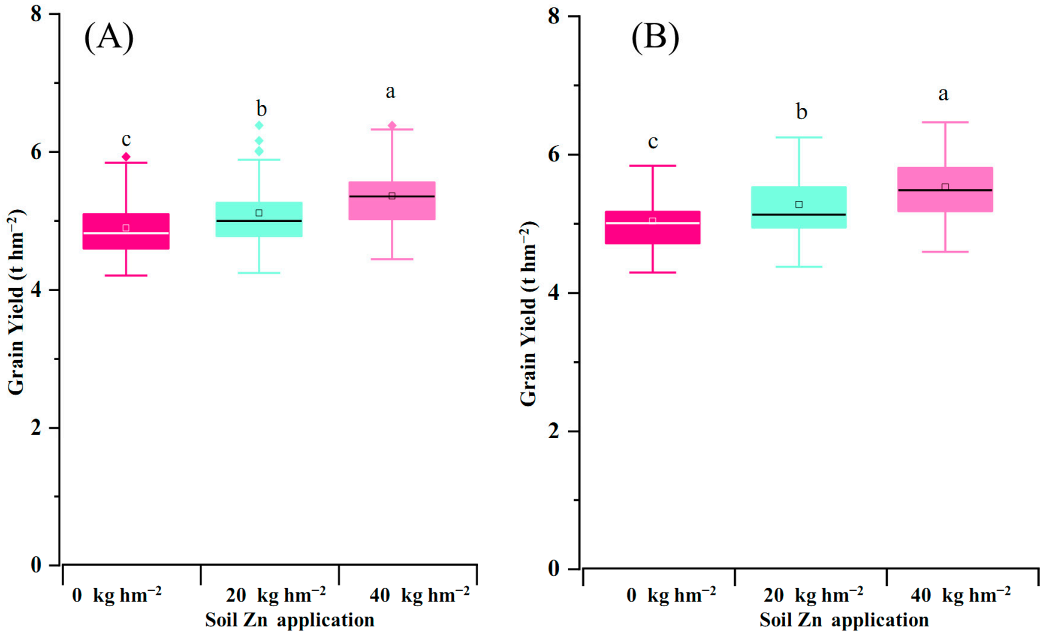

Fig S2. Grain yield of 20 wheat genotypes grown at three soil zinc levels (0 kg hm^{-2}, 20 kg hm^{-2}, and 40 kg hm^{-2} Zn by applying ZnSO4) under saline field conditions in 2020-2021 (A), and 2021-2022 (B) seasons.

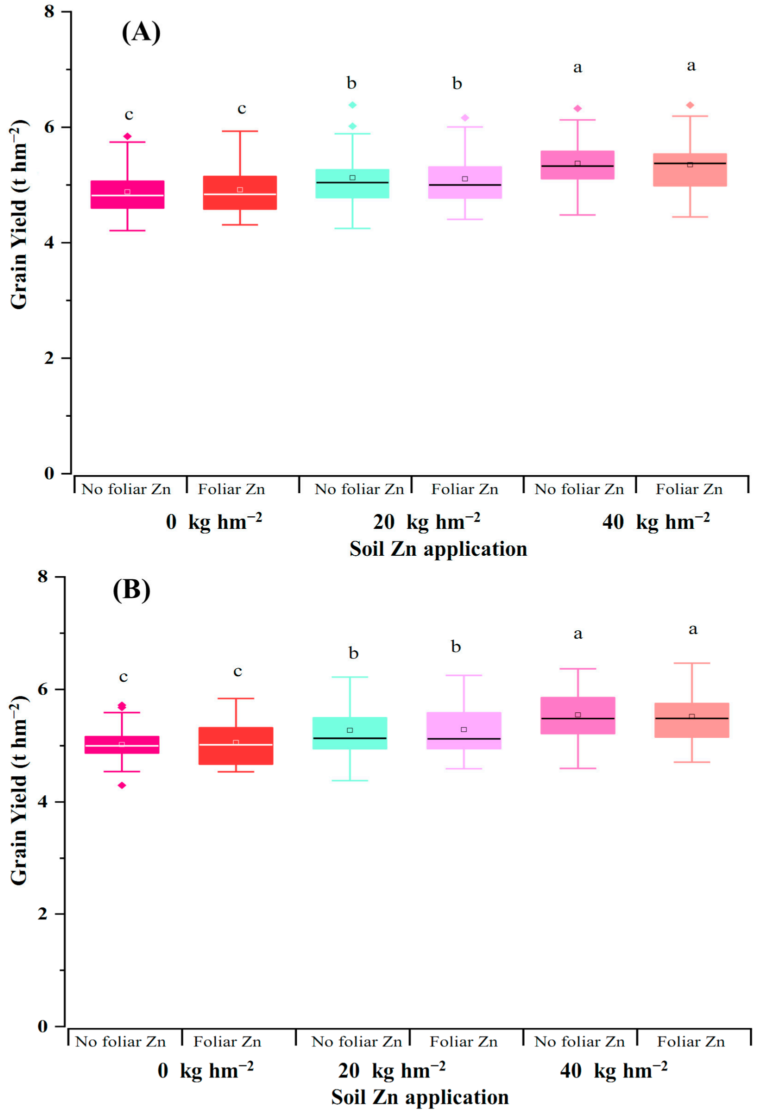

Fig S3. Average grain yield of 20 wheat genotypes grown at three soil zinc levels (0 kg hm^{-2}, 20 kg hm^{-2}, and 40 kg hm^{-2} Zn by applying ZnSO4) and two foliar zinc levels (No foliar application, and foliar application) under saline field conditions in 2020-2021 (A), and 2021-2022 (B) seasons.

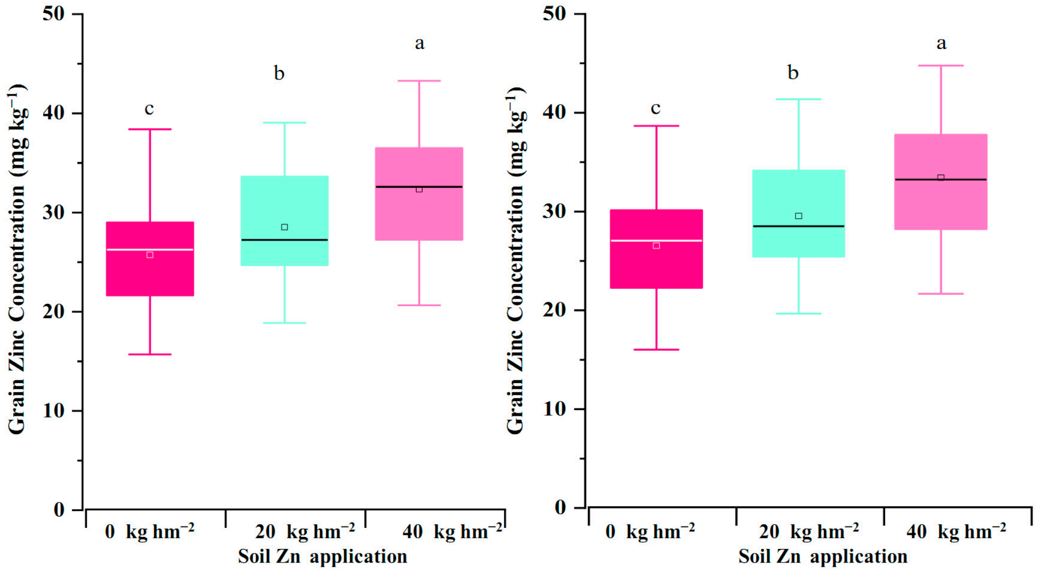

Fig S4. Grain zinc concentration of 20 wheat genotypes grown at three soil zinc levels (0 kg hm^{-2}, 20 kg hm^{-2}, and 40 kg hm^{-2} Zn by applying ZnSO4) under saline field conditions in 2020-2021 (A), and 2021-2022 (B) seasons.

Supplementary Table S2. Grain yield and grain zinc concentration of 20 wheat genotypes

No.	Wheat Genotype	Grain yield (t hm^{-2})		Grain zinc concentration (mg kg^{-1})	
		Average ± SD	Range	Average ± SD	Range
1	Line 4038	6.06 ± 0.21 a	5.74 - 6.37	19.88 ± 2.16 i	15.7 - 22.00
2	K356	5.00 ± 0.12 defghi	4.81 - 5.19	24.69 ± 3.40 ghi	18.1 - 29.17
3	Line 6782	5.18 ± 0.22 bcdefg	4.82 - 5.51	22.94 ± 3.15 hi	16.8 - 26.29
4	D4091	5.98 ± 0.29 a	5.54 - 6.47	23.03 ± 3.61 hi	17.6 - 28.51
5	B872	6.07 ± 0.28 a	5.84 - 6.37	26.06 ± 4.39 gh	19.5 - 32.88
6	Jiaomai 668	5.19 ± 0.29 bcdefg	4.83 - 5.70	28.57 ± 4.29 defgh	20.3 - 34.54
7	D8241	5.32 ± 0.31 bcde	4.84 - 5.88	28.26 ± 5.43 defgh	21.8 - 35.77
8	K427	4.79 ± 0.25 hij	4.41 - 5.17	27.27 ± 2.75 efgh	23.5 - 32.23
9	Yunong 416	4.94 ± 0.23 efghi	4.60 - 5.39	30.20 ± 4.43 cdefg	22.3 - 36.23
10	Line 1280	4.45 ± 0.16 j	4.21 - 4.72	38.80 ± 3.19 a	33.9 - 44.77
11	Zhengmai 366	5.54 ± 0.28 b	5.22 - 6.02	32.37 ± 3.13 bcde	26.5 - 37.05
12	Zhengmai 9405	5.49 ± 0.24 bc	5.09 - 5.82	34.74 ± 5.08 abc	27.7 - 43.72
13	Xinyang 75256	4.88 ± 0.21 fghi	4.60 - 5.30	31.57 ± 3.66 bcdef	26.7 - 38.07
14	Big grain No1	4.70 ± 0.31 ij	4.37 - 5.30	36.94 ± 5.26 ab	28.0 - 43.37
15	Taishan 4033	5.33 ± 0.23 bcd	5.03 - 5.72	34.41 ± 4.90 abc	26.3 - 42.75
16	Line 1051	4.85 ± 0.21 ghi	4.55 - 5.11	36.43 ± 3.83 ab	29.5 - 41.91
17	Line 6330	5.11 ± 0.33 cdefgh	4.70 - 5.62	33.18 ± 4.16 abcd	26.2 - 38.19
18	Hulutou	5.24 ± 0.35 bcdef	4.81 - 5.91	27.29 ± 3.34 efgh	21.5 - 31.35
19	Honghuomai	5.17 ± 0.27 bcdefgh	4.91 - 5.28	26.79 ± 2.66 efgh	23.5 - 32.49
20	Line 2884	4.82 ± 0.31 ghij	4.42 - 5.28	23.58 ± 2.52 hi	19.8 - 27.73

Note: different letters indicate a significant difference by Tukey HSD multiple comparsion (α=0.05).
